# Microsurgical clipping vs Woven EndoBridge (WEB) device for the management of unruptured wide-neck bifurcation aneurysms

**DOI:** 10.1007/s10143-022-01781-9

**Published:** 2022-04-11

**Authors:** Tatiana Chacón-Quesada, Dorothee Mielke, Veit Rohde, Silvia Hernández-Durán

**Affiliations:** Department of Neurosurgery, Universitätsmedizin Göttingen, Robert-Koch-Str. 40, 37075 Göttingen, Germany

**Keywords:** Intracranial aneurysm, Clipping, Woven EndoBridge device, Wide-neck aneurysms

## Abstract

The Woven EndoBridge device (WEB) was introduced in 2010 to treat wide-neck bifurcation aneurysms (WNBAs). Three landmark studies have been conducted to assess its safety and efficacy: WEBCAST, WEBCAST 2, and French Observatory Study. However, these studies have not compared its safety and efficacy to other treatment modalities. In this study, we compare WEB versus microsurgical clipping in the management of unruptured WNBA. We conducted a retrospective study of unruptured WNBA meeting the morphological criteria to be amenable for WEB treatment operated on at our institution. Surgical morbidity, mortality, and occlusion rates were assessed. We compared our results to those reported in the cumulative population of the three WEB landmark studies at 1 year. A total of 84 patients with 89 WNBA were included. The most common aneurysm location was the middle cerebral artery bifurcation (*n* = 67/89, 75%). No operative mortality was observed. Morbidity comprised small-vessel vasospasm (*n* = 1/89, 1%) resulting in hemiparesis vs. 3% morbidity for WEB (*p* = .324). All but one (*n* = 1/89, 1%) WNBA were completely occluded vs WEB occlusion rate of 53% at 1 year, statistically significantly worse (*p* < .001). In our analysis, we were not able to show superiority of WEB in terms of procedural morbidity in comparison to microsurgical clipping, defined as worsening in mRS. Microsurgical clipping achieves statistically significantly higher rates of complete aneurysm occlusion, thus posing the question of whether the WEB should be presented as a viable, comparable alternative to patients amenable to surgical treatment.

## Introduction

Wide-neck aneurysms (WNAs) are variably defined throughout the literature. The most widely utilized definition is based on a neck diameter ≥ 4 mm and/or a dome-to-neck ratio of < 2 [6, 12]. The particular anatomy of WNA makes their treatment either through surgical or endovascular modalities challenging [20]. On the one hand, surgical dissection of the neck and proper visualization of the parent and distal vessels can be obstructed [4]; on the other hand, coil migration and parent vessel occlusion can often ensue if these aneurysms are treated endovascularly [3, 9]. A meta-analysis by Fiorella et al. [4] revealed low occlusion rates of 39.8% and 52.5% for endovascular and surgical modalities in WNA, respectively. Regarding morbidity and mortality of patients with WNA, no specific data has been published. In a subgroup analysis of the Barrow Ruptured Aneurysm Trail (BRAT), Mascitelli et al. [10] reported that patients with WNA presented with worse clinical grading and had worse clinical outcomes at all timepoints when compared to those with narrow neck aneurysms. In cases of unruptured WNA, there is a paucity of data regarding morbidity and mortality.

Due to the complexity in their treatment, new endovascular techniques and devices have been developed for WNA. Among these is the Woven EndoBridge (WEB) device, an intrasaccular device designed to disrupt the intra-aneurysmal flow at the level of the neck and therefore induce aneurysmal thrombosis [13, 14, 17]. This device has been especially developed to treat wide-neck bifurcation aneurysms (WNBAs). Since its introduction in Europe in 2010, three landmark studies have been conducted to assess the safety profile and efficacy of the device for the treatment of ruptured and unruptured WNBA; WEBCAST [13], WEBCAST 2 [14] and the French Observatory Study [17]. While these studies have shown the use of the WEB device to be safe, with low periprocedural morbidity, they have not compared its safety and efficacy to other treatment modalities. Furthermore, cumulative analyses of the long-term effectiveness of the WEB device in terms of aneurysm occlusion have shown complete occlusion rates of approximately 50% [15, 16]. Thus, the goal of this study is to compare the clinical and anatomical results reported by Pierot et al. [15] in 2018, including midterm (1-year) follow-up of 169 WNBA in 168 patients treated with the WEB device in the WEBCAST, French Observatory, and WEBCAST-2 series to the results achieved in a retrospective cohort of morphologically similar aneurysms treated surgically.

## Materials and methods

### Patient population

We conducted a retrospective study of consecutive adult patients with unruptured WNBA who underwent microsurgical clipping at our institution between 2010 and 2020. The study was carried out in accordance with the 1964 Helsinki declaration, and internal review board (IRB) approval was obtained.

### Morphological inclusion and exclusion criteria

WEB has been approved for the treatment of WNBA in the middle cerebral artery (MCA) bifurcation, internal carotid artery (ICA) terminus, anterior communicating artery (ACom) complex, or basilar artery apex. Thus, we only included patients with aneurysms in these locations. As indicated by the WEB protocols, only unruptured WNBA with necks measuring between 2.5 and 8 mm, a size between 2.8 and 17 mm, and a dome-to-neck ratio > 1 and < 2 were included. Fusiform, multilobulated, or dissecting aneurysms were excluded.

### Surgical strategy

All patients underwent elective, standard pterional craniotomy, and microsurgical clipping with intraoperative indocyanide green (ICG) angiography and micro-Doppler ultrasound for verification of both parent and distal vessel patency. Postoperatively, all patients received a computed tomography angiography (CTA) to verify aneurysm occlusion and patency of the surrounding vessels, as well as to exclude perioperative infarctions or hemorrhages.

### Primary and secondary endpoints

Primary endpoint was complete aneurysm occlusion with no neck remnant, equivalent to a Raymond Roy Occlusion Classification (RROC) Class I for endovascularly treated lesions, as determined by CTA postoperatively.

Secondary endpoints were surgically related morbidity and mortality. Morbidity was defined analogous to Pierot et al. [15] in their pooled analysis of the WEBCAST [13], WEBCAST-2 [14], and French Observatory [17] trials: modified Ranking Scale (mRS) score > 2 if baseline ≤ 2 or mRS + 1 or more if baseline > 2. mRS was determined at last follow-up (FU). These primary and secondary endpoints in the surgical cohort were then compared to the ones reported by Pierot et al. [15] in the WEB cohorts.

Other secondary endpoints included surgically related adverse events (AEs), such as surgical site infections (SSI), seizures, vasospasm, postoperative hemorrhage/hematoma, intraoperative aneurysm rupture, or intraoperative clipping of parent and/or distal vessel.

### Statistical analysis

Continuous variables are reported as mean ± standard deviation (SD) or range. Categorical data are described both numerically (categorical total) and as a percentage of the analyzed population. The comparison between both treatment modalities, namely, our surgical cohort and the WEB pooled cohort, was carried out by means of chi square test. Significance was assumed at *p* < 0.05. Analyses were conducted using SPSS statistical software (IBM, Armonk, New York).

## Results

### Patients and aneurysms

A total of 84 patients harboring 89 WNBA aneurysms were included. Most patients (*n* = 63/84, 75%) were women. Mean age was 57 years (range: 38–79). Most patients (*n* = 56/84, 67%) had preoperative mRS 0; *n* = 20/84, 24% had mRS 1; *n* = 6/84, 7% had mRS 2, and *n* = 2/84, 2% had mRS 4. One of the patients with mRS 4 was a 35-year-old female who had suffered a malignant MCA infarction resulting in a high-grade hemiparesis and aphasia. The WNBA was diagnosed during the stroke workup. Because she improved during neurological rehabilitation to the point of being able to ambulate with significant assistance, her relatives opted for WNBA treatment. Similarly, the second patient was a 79-year-old man who had had a stroke 10 years prior. During follow-up of his ICA stenosis, the WNBA was detected. While the patient required significant assistance in activities of daily living, he wished to have the WNBA treated.

The most common aneurysm location was MCA bifurcation (*n* = 67/89, 75%), followed by ACom complex (*n* = 15/89, 18%) and ICA terminus (*n* = 7/89, 8%). Mean neck size was 4.06 ± 1.35 mm, while mean dome size was 6.00 ± 2.18 mm.

### Aneurysm occlusion rate

**Fig. 1:**
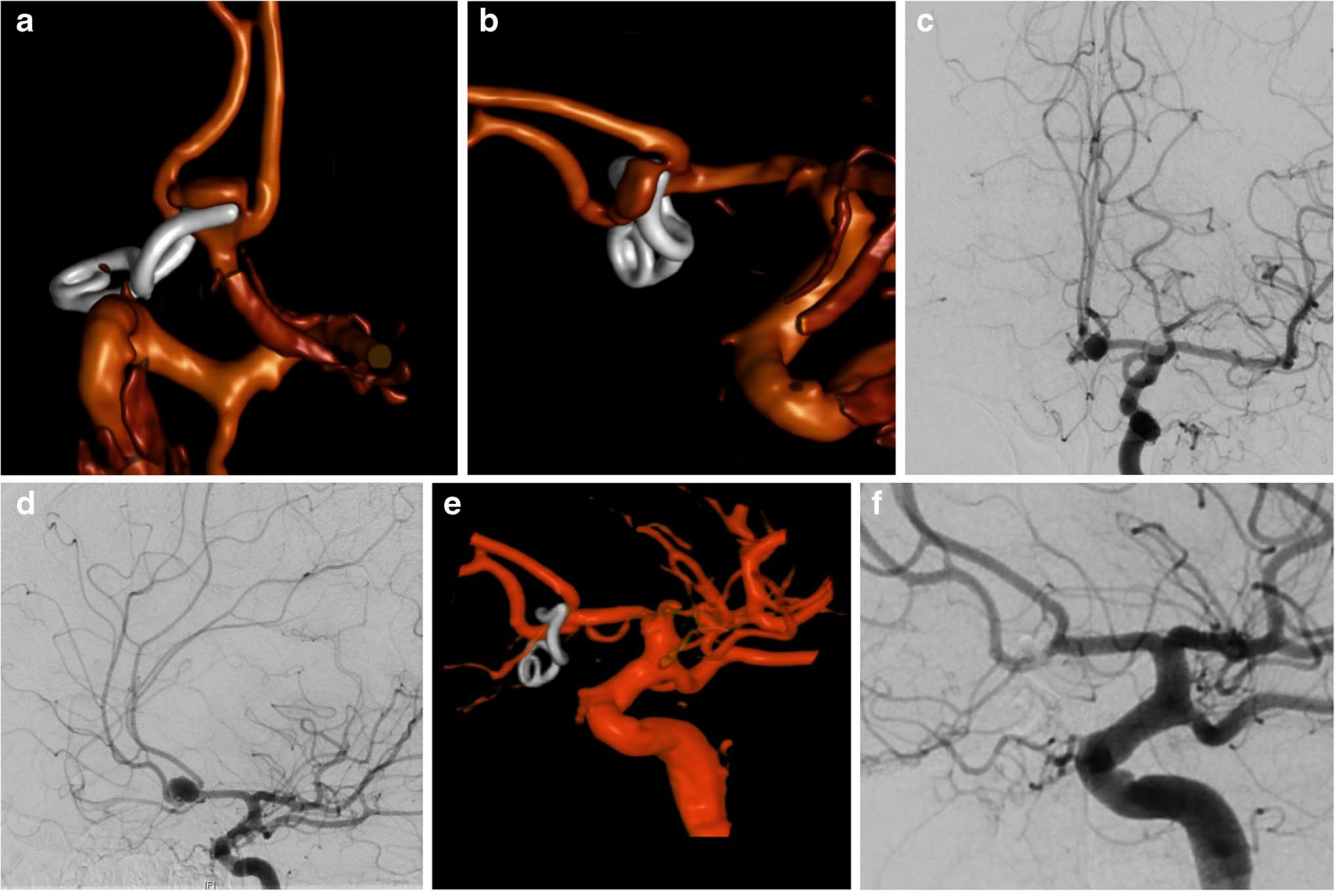
Clipped aneurysm showing neck remnant. Postoperative 3D reconstruction of CTA showing suspicion of a small neck remnant: **A** AP projection, **B** lateral projection, which was then confirmed by conventional angiography, **C** AP projection, **D** oblique projection. The remnant was subsequently coiled; **E** 3D reconstruction, **F** oblique projection, achieving complete occlusion

Aneurysm occlusion rate was 99%, with only one patient presenting incomplete microsurgical occlusion of the lesion. This was a 58-year-old woman with an ACom complex aneurysm with a maximum dome diameter of 9 mm and a neck size of 5 mm (Fig. 1A, B). The aneurysm was located between the two distal A2 branches. The surgical approach was a right pterional craniotomy. Visualization of the contralateral A2 was partially impeded by the aneurysm dome. The aneurysm was clipped with a curved bayonet tunnel clip, and occlusion was confirmed intraoperatively by both ICG angiography and ICG endoscopy. However, postoperative CTA showed suspicion of a small neck remnant, which was then confirmed by conventional angiography (Fig. 1C, D). The remnant was subsequently coiled (Fig. 1E, F). The patient had mRS 0 at discharge and was lost to follow-up.

No intraoperative aneurysm rupture occurred.

### Surgical adverse events, morbidity, and mortality

Mean follow-up in our cohort was 19 ± 24 months. At last follow-up, only one patient had surgically related morbidity, with a worsening mRS from 1 to 3, accounting for 1% morbidity. This was a 47-year-old woman who had suffered an aneurysmal subarachnoid hemorrhage (SAH) from a right MCA aneurysm. In the initial work-up, multiple aneurysms were detected, including two unruptured left-sided ones in the M1 and at the MCA bifurcation. Both were surgically approached through a left-sided standard pterional craniotomy 4 months after SAH, and the MCA bifurcation one was included in this study. It had a dome size of 6 mm, with a 4-mm neck and was completely occluded. Postoperatively, the patient exhibited right-sided hemiparesis. Work-up with CTA, CT perfusion, and magnetic resonance imaging (MRI) ruled out surgical occlusion of distal branches and infarction. She was thus treated for small-vessel vasospasm with slight improvement of her motor function. She was discharged to a rehabilitation facility 10 days after surgery. At 108 months follow-up, she was able to ambulate independently, but still required assistance in some activities of daily living (mRS 3).

Other surgical AE included SSI in *n* = 3/84, 4%; transient vasospasm with no clinical sequelae in *n* = 6/84, 7%; and single, postoperative seizure with no clinical sequelae in *n* = 4/84, 5%. No mortality (*n* = 0/84, 0%) was observed in our cohort.

### WEB or clip?

As illustrated in Table 1, our analysis did not show a statistically significant superiority of WEB vs microsurgical clipping in terms of procedural morbidity and mortality. However, microsurgical clipping achieved a statistically significantly higher rate of complete aneurysm occlusion, equivalent to a RROC Class 1 (*p* < 0.001).

**Table 1:** Comparison of primary and secondary endpoints between the surgical and the WEB cohorts

Variable	Surgical cohort	WEB cohort (Pierot et al. 2018)	*p*
Complete occlusion (*n*, %)	88/89 (99%)	81/169 (53%)	< 0.001*
Mortality (*n*, %)	0/84(0%)	0/168 (0%)	1.000
Morbidity (*n*, %)	1/84 (1%)	5/168 (3%)	0.3224

^*^Denotes statistical significance

## Discussion

Our study revealed comparable procedure-related morbidity in clipped unruptured WNBA and those treated with WEB device. For the purposes of this study, we employed the same definition of morbidity than the authors in the WEB cumulative studies, namely, a drop in mRS score. However, we do not believe that this definition fully reflects procedure-related adverse events. Adverse effects associated with microsurgical clipping included new-onset seizures, surgical site infections, and transient vasospasms with no permanent sequelae, which are not reflected in the mRS, thus underrepresenting the potential complications of this treatment modality. Contrarily, the adverse events reported by Pierot et al. [15] included 24/168, 14.4% thromboembolic events, out of which five went on to carry sequelae for the patients. Furthermore, intracranial hemorrhage occurred in one case, and intraprocedural rupture was documented in two cases, as opposed to no aneurysmal rupture in our surgical cohort.

Given that the majority of patients evaluated in the cumulative series by Pierot et al. [15] and in our own surgical cohort had unruptured intracranial aneurysms (UIAs), we believe that other tools or scales to assess outcome should be included. Quality of life (QoL) is an important variable to evaluate when treating UIA. A study performed by Zhai et al. [21] revealed that patients with UIA undergoing endovascular treatment had a decreased health-related quality of life (HRQoL) when compared to the general population. Conversely, the HEAT trial secondary study on HRQoL after endovascular coiling of UIA revealed that a subset of patients had improvements in some physical and emotional components of QoL after treatment [1]. While data is scarce regarding microsurgical clipping and HRQoL, Solheim et al. [19] showed that microsurgical clipping and endovascular coiling did not differ on their impact on patients’ HRQoL when undergoing treatment for UIA. The authors believe that evaluating the impact of WEB-assisted treatment of UIA on the patients’ HRQoL could be useful for future patient counseling and physician decision-making when selecting treatment modalities for UIA. Nevertheless, the most relevant finding of our study was the significantly higher aneurysm occlusion rate in the microsurgical group. In our series, the occlusion rate was 99%, with just a patient requiring retreatment. This is in line with previous studies. In a meta-analysis of microsurgical vs endovascular treatment of unruptured MCA aneurysms, Alreshidi et al. [2] demonstrated a statistically significantly higher occlusion rate through the surgical treatment modality with otherwise clinical equipoise. Similarly, Fiorella et al. [4] reported higher rates of WNBA occlusion in patients undergoing microsurgical clipping when compared to those treated endovascularly. Furthermore, Goertz et al. [5] carried out a similar comparison between WEB-Device and microsurgical clipping for WNBA of the anterior circulation. Like in our cohort, they reported higher occlusion rates for clipped aneurysms when compared to the WEB/endovascular arm. Contrary to their report, our study found a similar safety profile when comparing morbidity and mortality.

On the other hand, Pierot et al. [15] reported a success rate 96.4% (163/169). Among aneurysms treated with WEB, adjunctive devices were necessary in 12/163 (7.4%) aneurysms, thus yielding a relatively high rate of insufficient treatment through WEB alone. Furthermore, at 1 year follow-up, complete occlusion was achieved in just 81/153 (52.9%) aneurysms. While the partially occluded aneurysms remained stable and without clinical significance at mid-term follow-up, they represent a higher exposure to ionizing radiation for the patients, who must undergo follow-up angiographies [7] and eventually retreatment, with all the periinterventional risks that retreatment might carry.

The importance of complete occlusion in UIA has not been conclusively established yet and is one of the controversies in modern vascular neurosurgery [18]. On the one hand, data from ruptured aneurysms presenting with SAH has shown that the degree of aneurysm occlusion after treatment strongly correlates with the risk of rerupture. The Cererbral Aneurysm Rerupture After Treatment (CARAT) study [8] analyzed 1001 patients with SAH and found a statistically significant correlation between aneurysm occlusion rate and risk of rerupture. On the other hand, Munich et al. [11] demonstrated that patients with UIA with residual necks treated endovascularly by either coiling or stent-assisted coiling had a very low risk of rupture (0.6%), whereas those with ruptured aneurysms had a higher risk of rerupture from a neck remnant (3.4%). Thus, the natural history and true relevance of neck remnants after treatment with WEB device remains to be elucidated.

### Limitations

Due to the retrospective nature of the surgical arm of this study, our results must be interpreted with caution. It is possible that selection bias was present, since patients were selected for clipping based upon numerous patient and aneurysmal characteristics that might be contributed to more favorable outcomes. Additionally, all surgically treated patients underwent clipping at a tertiary referral center; this might underrepresent the real-life morbidity of patients with WNBA treated microsurgically and not be representative of general cerebrovascular surgical practice.

## Conclusions

Our analysis did not show superiority of the WEB device in terms of procedural morbidity in comparison to microsurgical clipping. Furthermore, microsurgical clipping achieved statistically significantly higher rates of complete aneurysm occlusion. The significance of complete occlusion in UIA remains a matter of debate, but until the natural history of neck remnants after WEB has been extensively investigated, the question of whether the WEB device should be presented as a viable, comparable alternative to patients amenable to surgical treatment is unanswered.

## Data Availability

Pertinent data are presented in the manuscript. Raw data can be made available at request pseudonymized.

## Code availability

N/A.

## References

[1] Abi-Aad KR, Rahme RJ, Syal A, Patra DP, Hudson M, Richter KR, Ward JD, Knis J, Nak Y, Turcotte E, Welz ME, Winter JD, Krishna C, Chong B, Bendok BR (2021). Quality of life of patients with unruptured intracranial aneurysms before and after endovascular coiling: a HEAT trial secondary study and systematic review of the literature. World Neurosurg.

[2] Alreshidi M, Cote DJ, Dasenbrock HH, Acosta M, Can A, Doucette J, Simjian T, Hulou MM, Wheeler LA, Huang K, Zaidi HA, Du R, Aziz-Sultan MA, Mekary RA, Smith TR (2018). Coiling versus microsurgical clipping in the treatment of unruptured middle cerebral artery aneurysms: a meta-analysis. Neurosurgery.

[3] Cloft HJ, Joseph GJ, Tong FC, Goldstein JH, Dion JE (2000). Use of three-dimensional Guglielmi detachable coils in the treatment of wide-necked cerebral aneurysms. AJNR Am J Neuroradiol.

[4] Fiorella D, Arthur AS, Chiacchierini R, Emery E, Molyneux A, Pierot L (2017). How safe and effective are existing treatments for wide-necked bifurcation aneurysms? Literature-based objective performance criteria for safety and effectiveness. Journal of NeuroInterventional Surgery.

[5] Goertz L, Liebig T, Pennig L, Roman Laukamp K, Timmer M, Brinker G, Schlamann M, Goldbrunner R, Dorn F, Krischek B, Kabbasch C (2021). Woven endobridge embolization versus microsurgical clipping for unruptured anterior circulation aneurysms: A propensity score analysis. Neurosurgery.

[6] Hendricks BK, Yoon JS, Yaeger K, Kellner CP, Mocco J, de Leacy RA, Ducruet AF, Lawton MT, Mascitelli JR (2020). Wide-neck aneurysms: systematic review of the neurosurgical literature with a focus on definition and clinical implications. J Neurosurg JNS.

[7] Ihn Y-K, Kim B, Woong Jeong H, Hyun Suh S, Dong Won Y, Lee Y-J, Joon Kim D, Jeon P, Ryu C-W, Suh S, Seob Choi D, Sung Choi S, Heum Kim S, Soo Byun J, Rho J, Song Y, Sang Jeong W, Hong N, Hyun Baik S, Jin Park J, Mee Lim S, Kim J-J, Yoon W (2021) Monitoring radiation doses during diagnostic and therapeutic neurointerventional procedures: multicenter study for establishment of reference levels Ihn YK et al. Reference Doses for Interventional Neuroradiology Procedures in Korea. 10.5469/neuroint.2021.00437 10.5469/neuroint.2021.00437 PMC8561028 34695909

[8] Johnston SC, Dowd CF, Higashida RT, Lawton MT, Duckwiler GR, Gress DR (2008). Predictors of rehemorrhage after treatment of ruptured intracranial aneurysms: the Cerebral Aneurysm Rerupture After Treatment (CARAT) study. Stroke.

[9] Lodi YM, Latorre JG, El-Zammar Z, Swarnkar A, Deshaies E, Fessler RD (2012). Stent assisted coiling of the ruptured wide necked intracranial aneurysm. J NeuroInterv Surg.

[10] Mascitelli JR, Lawton MT, Hendricks BK, Nakaji P, Zabramski JM, Spetzler RF (2019). Analysis of wide-neck aneurysms in the barrow ruptured aneurysm trial. Clin Neurosurg.

[11] Munich SA, Cress MC, Rangel-Castilla L, Sonig A, Ogilvy CS, Lanzino G, Petr O, Mocco J, Morone PJ, Snyder KV, Hopkins LN, Siddiqui AH, Levy EI (2018). Neck remnants and the risk of aneurysm rupture after endovascular treatment with coiling or stent-assisted coiling: much ado about nothing?. Neurosurgery.

[12] Park HS, Kwon SC, Park ES, Bum Park J, Kim MS (2019). A new definition for wide-necked cerebral aneurysms. J Cerebrovasc Endovasc Neurosurg.

[13] Pierot L, Costalat V, Moret J, Szikora I, Klisch J, Herbreteau D, Holtmannspötter M, Weber W, Januel A, Liebig T, Sychra V, Strasilla C (2016). Safety and efficacy of aneurysm treatment with WEB: results of the WEBCAST study. J Neurosurg JNS.

[14] Pierot L, Gubucz I, Buhk JH, Holtmannspötter M, Herbreteau D, Stockx L, Spelle L, Berkefeld J, Januel AC, Molyneux A, Byrne JV, Fiehler J, Szikora I, Barreau X (2017). Safety and efficacy of aneurysm treatment with the WEB: results of the WEBCAST 2 study. Am J Neuroradiol.

[15] Pierot L, Moret J, Barreau X, Szikora I, Herbreteau D, Turjman F, Holtmannspötter M, Januel AC, Costalat V, Fiehler J, Klisch J, Gauvrit JY, Weber W, Desal H, Velasco S, Liebig T, Stockx L, Berkefeld J, Molyneux A, Byrne J, Spelle L (2018). Safety and efficacy of aneurysm treatment with WEB in the cumulative population of three prospective, multicenter series. J NeuroInterv Surg.

[16] Pierot L, Szikora I, Barreau X, Holtmannspoetter M, Spelle L, Herbreteau D, Fiehler J, Costalat V, Klisch J, Januel AC, Weber W, Liebig T, Stockx L, Berkefeld J, Moret J, Molyneux A, Byrne J (2021). Aneurysm treatment with WEB in the cumulative population of two prospective, multicenter series: 3-year follow-up. J NeuroInterv Surg.

[17] Pierot XL, Moret J, Turjman F, Herbreteau D, Raoult H, Barreau X, Velasco S, Desal H, Januel AC, Courtheoux P, Gauvrit JY, Cognard C, Molyneux A, Byrne J, Spelle L (2016). WEB French observatory. Am J Neuroradiol.

[18] Santiago-Dieppa DR, Zhou T, Pannell JS, Khalessi AA, Veznedaroglu E (2016). Controversies in vascular neurosurgery: aneurysm remnants. Controversies in Vascular Neurosurgery.

[19] Solheim O, Eloqayli H, Muller TB, Unsgaard G (2006). Clinical article quality of life after treatment for incidental, unruptured intracranial aneurysms. Acta Neurochir (Wien).

[20] Won SY, Seifert V, Dubinski D, Kashefiolasl S, Dinc N, Bruder M, Konczalla J (2021). Short- and midterm outcome of ruptured and unruptured intracerebral wide-necked aneurysms with microsurgical treatment. Sci Rep.

[21] Zhai X-D, Ma Y-J, Yu J-X, Wang C-X, Geng J-W, Xiang S-S, Wang J, Guan X, Li G-L, He C, Hu P, Zhang H-Q (2021). Health-related quality of life outcomes and influencing factors in patients with unruptured intracranial aneurysms after endovascular treatment. Qual Life Res.

